# Reprogramming rice leaves: another layer of senescence regulation

**DOI:** 10.1093/jxb/erac178

**Published:** 2022-08-11

**Authors:** Hilary Joan Rogers

**Affiliations:** Cardiff University, School of Biosciences, Sir Martin Evans Building, Museum Avenue, Cardiff CF10 3AX, UK

**Keywords:** ChIP seq, histone modifications, H3K9ac, leaf senescence, rice

## Abstract

This article comments on:

**Zhang Y, Li Y, Zhang Y, Zhang Z, Zhang D, Wang X, Lai B, Huang D, Gu L, Xie Y, Miao Y.** 2022. Genome-wide H3K9 acetylation level increases with age-dependent senescence of flag leaf in rice (*Oryza sativa*). Journal of Experimental Botany **73,**4696–4715.

**How does a leaf switch its gene expression from active photosynthesis to senescence, ending in death of the organ? Making sure this process is tightly regulated is especially important in a major crop such as rice where the remobilization occurring during senescence is critical to the development of the seed and ultimately crop yield. Zhang et al. (2022) now show that epigenetic reprogramming through genome-wide histone H3 acetylation (H3K9ac) has an important role to play in rice leaf senescence.**

The most obvious visible sign of leaf senescence is yellowing, caused by the ordered disassembly of the photosynthetic machinery and the breakdown of chlorophyll (Kuai *et al.*, 2018). Leaf senescence is therefore sometimes neglected as an end-of-life process in which metabolism shuts down, and all cells ultimately die. However, we know from a wealth of studies in model plants such as *Arabidopsis thaliana* (Breeze *et al.*, 2011; Guo *et al.*, 2021) that it is in fact a carefully regulated active developmental process in which transcript levels of suites of genes increase as well as decrease. The key function of senescence is in the remobilization of nutrients to other parts of the plant, crucially in monocarpic crops such as rice, to the developing seed (Lee and Masclaux-Daubresse, 2021). Although leaf senescence is a developmental process, it is also affected by environmental factors such as drought, cold, and other abiotic factors as well as pathogen attack. These signals are mediated by phytohormones: of particular importance are positive regulators: abscisic acid (ABA), ethylene, jasmonic acid (JA), salicylic acid (SA), and brassinosteroids, while cytokinin and gibberellin act as negative regulators of senescence, and the role of auxin is less clear (Ahmad and Guo, 2019). It is assumed that environmental signals are perceived at the cell surface and transduced through receptor-like kinases (RLKs) and mitogen-activated protein kinases (MAPKs), and an increasing number of RLKs and MAPKs are being associated with leaf senescence signalling (Ahmad and Guo, 2019). These in turn modulate the expression of transcription factors important for orchestrating the transcriptional shift during senescence (Fig. 1). Two important families of transcription factors have been associated with regulating transcription during senescence: the NAC and WRKY families, although MYB family transcription factors, also associated with stress responses, and transcription factors from other families, such as bHLH, C2H2, AP2/ERF, bZIP, and HB, are also involved in rice (Lee and Masclaux-Daubresse, 2021) and Arabidopsis (Balazadeh *et al.*, 2008). Importantly, these transcription factors act as networks with hubs responding to overlapping sets of signals (Hickman *et al.*, 2013).

**Fig. 1.**
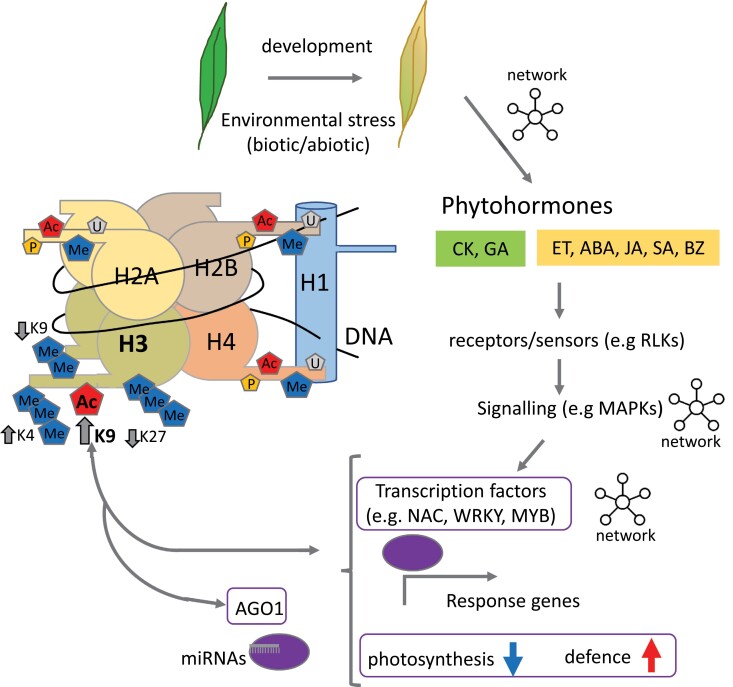
Senescence, seen as leaf yellowing, is regulated at many different levels. Networks of phytohormones (CK, cytokinins, GA, gibberellic acids, ET, ethylene, ABA, abscisic acid, JA, jasmonic acid, SA, salicylic acid, BZ, brassinosteroids) control the process to accelerate (yellow) or delay (green) progression of senescence. These are perceived via receptors, and signal, often via networks of MAPKs, to transcription factors. These in turn regulate response genes such as those involved in down-regulating photosynthesis and up-regulating defence and metabolism required for remobilization of nutrients to other parts of the plant such as developing seeds. This whole signalling network is also regulated by histone modifications. Different histone modifications: methylation (Me), acetylation (Ac), phosphorylation (P), and ubiquitination (U) up- (↑) or down- (↓) regulate gene expression either directly through association with genes for downstream processes or indirectly through regulation of transcription factors and the production of miRNAs. The signalling may also work in reverse such that transcription factors recruit enzymes required for histone modification.

An increasing number of developmental shifts and environmental responses in plants are being revealed to be under epigenetic control, and a key process is histone modification (Zhao *et al.*, 2019). Modifications can repress transcription such as the dimethylation of histone H3 at lysine 9 (H3K9me2) or can activate it such as acetylation of histone H3 at lysine 9 (H3K9ac) (Zhou *et al.*, 2010; Fig. 1). By applying ChIP-seq to map where modified histones bind across the genome, and comparing the pattern with changes in the transcriptome, it is now possible to obtain a dynamic profile of the effects of histone modification on gene expression.

Several histone marks have recently been associated with leaf senescence. These include the repressive mark H3K27me3 (Wang *et al.*, 2019), as well as H3K9ac and H3K4me3, both linked to active gene expression (Brusslan *et al.*, 2015). In Arabidopsis, H3K9ac was shown to be associated with genes whose expression rises during leaf senescence, indicating it may play a role in senescence regulation (Brusslan *et al.*, 2015). In this earlier study, a number of genes were found associated with the H3K9ac mark, but surprisingly not the widely accepted senescence marker *SAG12* which was instead associated with the H3K4me3 mark. H3K9ac has also been implicated in several other plant responses to their environment including de-etiolation (Charron *et al.*, 2009), and response to drought (Kim *et al.*, 2012), as well as in developmental reprogramming such as that occurring during flowering (Adrian *et al.*, 2010).

## Is binding to H3K9ac important for senescence progression?

Zhang *et al* (2022) have revisited the role of H3K9ac in an important crop—rice. Six time points were sampled from green mature leaves through to the beginning of visible senescence at the leaf tip to leaves that were obviously yellowing across the whole blade. The distribution of H3K9ac binding across each gene showed a similar pattern to that in Arabidopsis (Brusslan *et al.*, 2015), with most binding in intragenic and promoter regions. This conservation across taxonomically divergent species might suggest an early evolution of this epigenetic regulation and indeed H3K9ac is associated with actively transcribed genes across animals as well (Zhou *et al.*, 2010). Association with H3K9ac rose during senescence, indicating an increasing importance for this regulator as senescence progressed. The types of genes associated with a loss of H3K9ac binding are consistent with our understanding of senescence in which photosynthesis and biosynthesis shut down (Lee and Masclaux-Daubresse, 2021). Likewise, pathways most associated with an increase in H3K9ac binding included defence and metabolic processes, again associated with up-regulation during leaf senescence (Breeze *et al.*, 2011; Guo *et al.*, 2021). These findings confirm that, indeed, H3K9ac binding is consistent with a regulation of known senescence processes.

## How H3K9ac may affect transcription

Histone acetylation on lysine residues increases transcription by relaxing the chromatin structure. In Arabidopsis, this can occur in response to growth regulators, mediated via transcription factors. For example, under high auxin conditions, Arabidopsis bZIP11-related transcription factors bind to the promoters of auxin-responsive genes and, via an adaptor protein, they recruit histone acyltransferase complexes, resulting in upstream histone acetylation (Weiste and Dröge-Laser, 2014). Growth regulator signalling pathways were targeted by H3K9ac during rice leaf senescence (Zhang *et al.*, 2022), including the major stress- and senescence-related regulators: ABA, ethylene, and JA, as well as auxin and SA. In addition, transcription factors from >30 different families were also associated with changes in expression during leaf senescence and the H3K9ac mark. It will be interesting to see if further work can elucidate how the acetylation of histone H3 is modulated and whether similar or different mechanisms from that seen above for auxin regulation in Arabidopsis apply.

Zhang *et al.* (2022) also provide insights into a quantitative effect of the H3K9ac binding: in most highly expressed genes, binding was spread across a wider area; indeed, binding extended into the gene body as was also previously found in Arabidopsis (Brusslan *et al.*, 2015). Again, a clear mechanism for this is missing but may be important in understanding the fine-tuning of the effects on transcription.

## Dynamic layers of regulation

The sequence of different control points during senescence is critical to the ordered disassembly of chloroplasts and efficient remobilization of nutrients. In Arabidopsis microarray analysis identified the precise sequence of gene expression changes, showing that up-regulation of autophagy and reactive oxygen species were early events followed by an increase in the expression of genes related to ABA and JA signalling, all of which preceded the down-regulation of genes related to photosynthesis (Breeze *et al.*, 2011). In rice, down-regulation of photosynthesis also coincides with the first signs of leaf yellowing (Zhang *et al.*, 2022), and activation of OsPME1 involved in JA biosynthesis (Fang *et al.*, 2016) also starts early. This suggests conservation of at least some of the chronology of events between rice and Arabidopsis leaf senescence.

MiRNAs represent another layer of gene expression regulation that controls both developmental and environmental responses, and miRNA control of leaf senescence in Arabidopsis is now well established (Qin *et al.*, 2016). Many of the miRNA families involved in Arabidopsis leaf senescence are conserved in rice. Zhang *et al.* (2022) provide new evidence that miRNA synthesis may be under control of H3K9 acetylation. AGO1 is required for miRNA biosynthesis (Vaucheret, 2008), and both OsAGO1d expression and its association with H3K9ac increased during leaf senescence. An interaction between H3K9ac marks and miRNA regulation has been found in response to pathogens in tomato (Crespo-Salvador *et al.*, 2020), where expression of a WRKY transcription factor is associated with H3K9ac marks but is also miRNA regulated, and the miRNA structural gene is itself linked to H3K9ac marks in response to the pathogen. This interaction between epigenetic layers is worthy of further exploration to better understand the role of other histone marks, interactions with DNA methylation, and the possibility of coordinated control in the important regulatory pathways in leaf senescence.

## Conclusions and perspectives

Understanding how the different layers of epigenetic control mesh with each other and with growth regulator control and other signalling networks in regulating the initiation and progression of leaf senescence will open up new avenues for its potential manipulation to optimize crop productivity. Importantly, we also need to understand the operation of these networks during stress-induced senescence to mitigate the effects of a changing climate and pathogen landscape. Current approaches to manipulating leaf senescence have largely focused on growth regulators and transcription factors (Guo *et al.*, 2021), and encouragingly have been most successful in improving grain yield under stress (e.g. Peleg *et al.*, 2011). However, new tools are needed to improve food security and, by applying an improved understanding of epigenetic control, it may be possible to fine-tune senescence towards optimal grain production for the future.
